# Genotypic and phenotypic presentation of Glu89Gln mutation in Turkey

**DOI:** 10.1186/1750-1172-10-S1-P33

**Published:** 2015-11-02

**Authors:** Hacer Durmus, Zeliha Matur, Murat Mert Atmaca, Mehves Poda, Arman Cakar, Piraye Serdaroglu-Oflazer, Feza Deymeer, Yesim Parman

**Affiliations:** 1 Istanbul University, Istanbul Medical Faculty, Neurology Department, 34093, Istanbul, Turkey; 2 Istanbul Bilim University, Medical Faculty, Neurology Department, 34394, Istanbul, Turkey; 3 Istanbul University, Institute for Experimental Medical Research, Department of Genetics, 34093, Istanbul, Turkey

## Background

Transthyretin-related familial amyloid polyneuropathy (TTR-FAP) is an autosomal dominant disorder caused by mutations of the transthyretin (TTR) gene. More than 100 different mutations of the transthyretin gene are identified worldwide, but still the first described Val30Met is the most common one. The mutant amyloidogenic transthyretin protein causes systemic accumulation of amyloid fibrils that results in organ dysfunction and death. TTR-associated FAP is a progressive and fatal disease if left untreated and should be considered in the differential diagnosis of any patient with a progressive polyneuropathy, especially with an accompanying autonomic involvement.

## Patients and methods

We studied clinical, electrophysiological, histopathological, and genetic characteristics in five patients from two unrelated families with Glu89Gln mutation in TTR gene. Genetic testing was performed upon written informed consent to all patients according to the Declaration of Helsinki.

## Results

Mean age of onset was 51.8±9.14 (ranges 37-52) years. Three patients (two male) with Glu89Gly mutation had carpal tunnel syndrome (CTS) as presenting symptom. Two of them had asymmetric parasthesias in feet at presentation. Index patient had vocal cord involvement and spinal stenosis due to amyloid accumulation, which are rare manifestations of TTR-FAP. Vocal cord paralysis caused severe obstructive sleep apnea in this patient; he was treated with BIPAP successfully. All patients, except one patient in very early stage of disease had cardiomyopathy and autonomic involvement. One patient underwent intestinal biopsy due to severe diarrhea and biopsy was compatible with intestinal amyloidosis. Two patients died during follow-up at age 60 and 57 due to the systemic involvement. Patients were followed-up under Tafamidis meglumine treatment.

## Conclusion

Our cases suggest that Glu89Gln mutation could present with bilateral CTS and cause some atypical manifestations such as asymmetrical onset, vocal cord involvement and spinal stenosis.

